# Clinical Applications of Circulating Tumor Cells and Circulating Tumor DNA as a Liquid Biopsy Marker in Colorectal Cancer

**DOI:** 10.3390/cancers13184500

**Published:** 2021-09-07

**Authors:** Isabel Heidrich, Thaer S. A. Abdalla, Matthias Reeh, Klaus Pantel

**Affiliations:** 1 Center of Experimental Medicine, Department of Tumor Biology, University Medical Center Hamburg-Eppendorf, 20246 Hamburg, Germany; i.heidrich@uke.de; 2 Department for Operative Medicine University, University Medical Center Hamburg-Eppendorf, 20246 Hamburg, Germany; thaer.abdalla@uksh.de (T.S.A.A.); m.reeh@uke.de (M.R.)

**Keywords:** colorectal cancer, liquid biopsy, biomarker

## Abstract

**Simple Summary:**

Colorectal cancer is one of the most frequent malignant tumors worldwide and the spread of tumor cells through the blood circulation followed by the colonization of distant organs (“metastases”) is the main cause of cancer-related death. The blood is, therefore, an important fluid that can be explored for diagnostic purposes. Liquid biopsy is a new diagnostic concept defined as the analysis of circulating tumor cells or cellular products such as cell-free DNA in the blood or other body fluids of cancer patients. In this review, we summarize and discuss the latest findings using circulating tumor cells and cell-free DNA derived from tumor lesions in the blood of patients with colorectal cancer. Clinical applications include early detection of cancer, identification of patients with a high risk for disease progression after curative surgery, monitoring for disease progression in the context of cancer therapies, and discovery of mechanisms of resistance to therapy.

**Abstract:**

Colorectal cancer (CRC) is the third most commonly diagnosed cancer worldwide. It is a heterogeneous tumor with a wide genomic instability, leading to tumor recurrence, distant metastasis, and therapy resistance. Therefore, adjunct non-invasive tools are urgently needed to help the current classical staging systems for more accurate prognostication and guiding personalized therapy. In recent decades, there has been an increasing interest in the diagnostic, prognostic, and predictive value of circulating cancer-derived material in CRC. Liquid biopsies provide direct non-invasive access to tumor material, which is shed into the circulation; this enables the analysis of circulating tumor cells (CTC) and genomic components such as circulating free DNA (cfDNA), which could provide the key for personalized therapy. Liquid biopsy (LB) allows for the identification of patients with a high risk for disease progression after curative surgery, as well as longitudinal monitoring for disease progression and therapy response. Here, we will review the most recent studies on CRC, demonstrating the clinical potential and utility of CTCs and ctDNA. We will discuss some of the advantages and limitations of LBs and the future perspectives in the field of CRC management.

## 1. Introduction

Colorectal cancer (CRC) is the 4th leading cause of cancer-related death worldwide [1]. It is expected that the global burden of CRC will increase by 60% by 2030 making CRC a major global health problem [2].

In recent decades, there has been remarkable progress in the management of CRC. Starting with the implementation of national screening programs for the early detection of CRC [3], as well as the improvement of the surgical technique with the introduction of total mesorectal excision for rectal cancer and complete mesocolic excision for colon cancer, where sharp dissection along the embryological planes increases lymph node yields, subsequently improving staging and survival [4,5]. In addition, different neoadjuvant and adjuvant biological, chemotherapeutic, and radiotherapeutic strategies have been developed in the last two decades to improve survival and overall outcome in patients with CRC. The goal of these strategies is not only to reach resectability but also to increase local and systemic tumor control [6]. However, the overall outcome is still limited; currently, the overall 5-year survival is 65% [1,7]. Tumor recurrence or formation of distant metastases still occurs in 20% of the patients despite proper treatment and is the leading cause of death in these patients [8].

It is well known that the prognosis of CRC is dependent on the tumor stage at diagnosis. The most common used system for tumor classification is the AJCC TNM staging system, which uses anatomical parameters to discriminate patients into different groups with variable survival outcomes [9]. However, CRC is a very heterogeneous disease in respect to the clinical and tumor-related features, resulting in overwhelming differences in the course of disease and treatment responses. These differences in CRC complicate prognostication and guidance for optimal timing and treatment selection at an individual level [10]. Therefore, new non-invasive biomarkers are needed to personalize therapies and to prevent both under and overtreatment of CRC patients.

More than 10 years ago, the term liquid biopsy (LB) has been introduced by Pantel and Alix-Panabieres [11] as a minimally invasive way for tissue sampling, which allows for analysis of tumor cells or tumor cell products (e.g., cell-free circulating nucleic acids (ctDNA, cfRNA), extracellular vesicles, or proteins) released from primary or metastatic tumor lesions into blood or other body fluids [12]. Here, we discuss recently published reports on CTCs and ctDNA (within the last five years) because these are the most prominent LB markers [12], and we restricted our review to studies in patients with colorectal carcinoma as one of the most common solid tumors worldwide [13]. Following a brief introduction of CTC and ctDNA technologies, we will focus on the current clinical applications of these biomarkers, including early detection, risk assessment, and monitoring of cancer therapies.

## 2. Technologies for CTC and ctDNA Analyses

Before discussing the clinical applications, we would like to give a brief overview of the methods used for the enumeration and characterization of CTCs and ctDNA.

### 2.1. CTCs

Working with CTCs includes the following three analytical steps: enrichment, detection, and analysis. Enrichment includes label-dependent approaches based on antibodies used for positive or negative enrichment of CTCs as well as label-independent technologies (e.g., size exclusion by microfiltration, in which blood is passed through filters with small pores or microfluidic chips calibrated to capture CTCs). Effective enrichment exploits differences between tumor cells and normal blood cells, such as differential expression of tumor-associated cell surface proteins (e.g., EpCAM, mucin-1, HER2, or epithelial growth factor receptor (EGFR)) or distinct physical properties (e.g., larger size or reduced deformability) of tumor cells [14]. In contrast, negative enrichment approaches enrich CTCs by the depletion of normal blood cells that are removed by antibodies against antigens expressed on leukocytes or circulating endothelial cells [15]. Besides the capture of single tumor cells, CTC clusters have attracted recent attention [13,16].

CTCs can be identified by the use of specific tumor-associated or tissue-specific proteins such as keratins in patients with carcinomas. However, keratins (and other epithelial markers) can be downregulated or lost during an epithelial-mesenchymal transition (EMT) of the tumor cells, which can lead to false-negative findings [17]. Therefore, new markers are being sought out that are neither downregulated during EMT nor expressed on normal blood cells.

In the last decade, individual CTCs or CTC clusters could be analyzed downstream at the DNA, RNA, or protein level. Separation of individual CTCs can be achieved by manual micromanipulation or automated DEP array technology, but usually, a sufficiently high initial CTC concentration is required [18]. The whole genome amplification (WGA) method has been used to perform DNA analysis on a single cell level to generate a sufficient amount of DNA for subsequent sequencing analysis. However, WGA causes bias; thus, new methods avoiding WGA are currently being developed. In addition to RNA sequencing, multiplex real-time polymerase chain reaction can already provide some insights into the heterogeneity of CTCs [19]. In addition to protein-level analysis using immunostaining, new multiplex proteomics approaches are under development.

In addition to descriptive methods, there are functional CTC analyses, such as epithelial immune SPOT, which is based on the measurement of secreted proteins by live CTCs after short-term culture. In patients with extremely high numbers of CTCs (usually > 100/mL of blood), the functional properties of CTCs can be further investigated by establishing long-term cell cultures/cell lines or CTC-derived xenograft models [20,21]. These models provide unique insights into the functional properties of CTCs but the success rate of establishing these models needs to be improved to use them for drug screening in clinical trials or decision making for individual patients in advanced disease stages.

### 2.2. ctDNA

Circulating free DNA (cfDNA) is released by both normal and tumor cells to the blood circulation mainly through cellular necrosis and apoptosis but active secretion through EVs may also play a role [22]. cfDNA consists mostly of 166 bp, which is consistent with the length of a DNA fragment wrapped around a nucleosome. In cancer patients, a small portion of cfDNA (usually 0.01–5%) is shed into the blood by tumor cells; this is called ctDNA (ctDNA, which is shed from tumor cells, represents a small portion of cfDNA (usually 0.01–5%)) [14]. ctDNA is cleared soon after entering the circulation due to its short half-life of two hours, allowing for non-invasive real-time tumor monitoring [23,24]. The molecular biological analysis allows for the identification and characterization of ctDNA. The following paragraph briefly delineates the types of ctDNA analyses depending on the objective of the planned investigation.

Plasma DNA can be analyzed by approaches targeting specific tumor-associated genes (e.g., mutations in the EGFR gene in non-small cell lung carcinoma (NSCLC)) or non-targeted screening approaches such as array CGH, whole-genome sequencing, or exome sequencing) [25,26]. In general, targeted approaches have higher analytical sensitivity than non-targeted approaches, but there are strong efforts to improve the detection limits of non-targeted approaches [27,28]. Ultrasensitive methods have been developed for the detection of minute amounts of 0.01% or less ctDNA in blood plasma (e.g., DELPHI, BEAMing Safe-SeqS, TamSeq, CAPP-Seq, and digital PCR) [12,29]. In addition to mutation analysis, reliable assays for assessing epigenetic changes such as DNA methylation have been developed in recent years for blood testing in several types of solid tumors including CRC [30,31,32].

## 3. Clinical Applications of Circulating Tumor Cells (CTCs)

**Figure 1:**
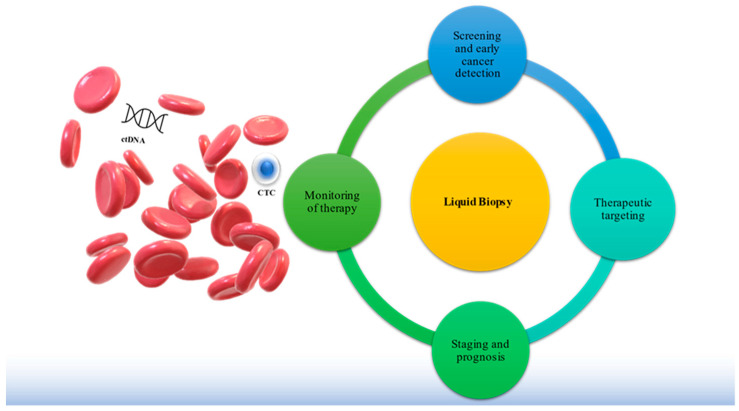
Clinical applications of liquid biopsies. Clinical hallmark applications of liquid biopsy (LB): (1) screening and early cancer detection, (2) therapeutic targeting, (3) staging and prognosis, and (4) monitoring of therapy. LB allows for the portrayal of the entire disease by using blood-based tumor-specific biomarkers, such as circulating tumor cells (CTCs) and circulating tumor DNA (ctDNA) released from all metastatic or primary tumor sites to provide comprehensive and real-time information on tumor cell evolution, therapeutic targets, and mechanisms of resistance to therapy.

CTCs have the potential to extravasate and seed metastases in distant organs, which is the most common reason for cancer-related death in CRC and other solid tumors. CTC analysis has the potential to be used as a biomarker for tumor detection, prognostication, therapy monitoring, and to tailor appropriate individualized treatments (Figure 1). The following chapter illustrates the latest developments of CTC-based clinical studies in CRC [33].

### 3.1. Early Detection of Cancer

The ideal screening tool should be reproducible and efficient with high sensitivity and specificity. The detection of CTCs in CRC is still infrequent and limited. According to Bork et al., the detection of CTCs in nodal negative CRC (UICC stage I–II) is as low as 9% [34,35]. In another study, the detection of CTCs using Cellsearch^®^ in CRC (UICC stage I–IV) was 45% [26]. Therefore, the utility of CTC-based screening using Cellsearch^®^ is still rather challenging and still not applicable (Table 1).

In 2018, Tsai et al. reported for the first time that CTCs could be used for early cancer detection. This prospective study was conducted on 620 subjects including 438 with precancerous lesions or CRC (UICC stage I–IV) and 182 healthy controls. CTC detection was performed using the Cellmax platform, which uses a microfluidic anti-EpCAM-antibody-coated biochip. In precancerous lesions, CTCs showed a sensitivity of 76.6%, a specificity of 97.3%, and an area under the curve (AUC) of 0.84. In patients with CRC, CTC showed a sensitivity of 86.9%, specificity of 97.3%, and AUC of 0.88 [42]. Despite the promising results, larger validation studies are needed before the implementation of CTC-based screening using Cellmax in CRC.

### 3.2. Prediction of Treatment Response and Survival

Mesenteric venous blood compartments of patients with CRC harbor more CTCs than the peripheral blood, which might be explained by the fact that viable CTCs can home to the liver, frequently leading to liver metastasis in CRC [43]. In advanced CRC, several studies have shown that CTC count before and during treatment predicts PFS and OS and provides additional information beyond CT imaging [44,45,46], whereas surgical resection of metastases immediately lowers CTC levels [47]. Patients with elevated CTC count, even when classified as responders by CT imaging, showed significantly shorter survival (Table 1) [35].

In patients who underwent curative resection (stage III) followed by FOLFOX chemotherapy, CTC count predicted relapse after chemotherapy [48]. In nonmetastatic CRC, preoperative CTC detection is an independent prognostic marker [34], and CTC count correlated with reduced DFS [49]. Thus, CTC detection could help select high-risk stage II CRC candidates for adjuvant chemotherapy [50,51]. Interventional studies are now needed to assess whether stage II patients with CTCs benefit from chemotherapy. Recently, Aranda et al. evaluated whether CTC counts may be a useful non-invasive biomarker to assist with the selection of patients for intensive therapy with FOLFOXIRI-bevacizumab. This combination is more effective than FOLOFOX plus bevacizumab but is not widely used because of concerns about toxicity and so far a lack of predictive biomarkers. In their phase III study in patients with previously untreated, unresectable metastatic CRC, Aranda et al. found that first-line FOLFOXIRI-bevacizumab significantly improved PFS compared with FOLFOX-bevacizumab in patients with metastatic CRC who presented with ≥3 CTCs at baseline [52].

Thus, CTC enumeration can contribute to the identification of a high-risk group of CRC patients who might profit from more intense therapy.

### 3.3. Molecular and Functional Characterization of CTCs

KRAS, BRAF, and PIK3CA mutations are important determinants of CRC patients’ response to targeted therapies. For example, blocking EGFR signaling by an antibody therapy in CRC is inefficient in patients with mutated KRAS tumors, which provide a stimulatory signal downstream of EGFR. In-depth analysis of individual CTCs from patients with CRC revealed the striking heterogeneity of KRAS status within and between patients [53,54], and the occurrence and concordance of these mutations in metastatic CRC may vary between primary tumors, CTCs, and metastatic tumors [54,55,56]. When KRAS mutations in CTCs from patients with metastatic CRC were examined throughout disease progression and compared with their corresponding primary tumors, CTCs had different KRAS mutations during treatment [57]. Thus, CTCs are promising markers for evaluating and predicting treatment response in patients with rectal cancer superior to carcinoembryonic antigen [58]. Liquid biopsy analyses might also lead to the discovery of new targets. For example, the comparative analyses of blood from healthy controls, patients with polyps and adenomas, and cancer patients revealed that lncRNA SNHG11 might serve as a novel therapeutic target in CRC [59].

Among cancer therapies, the new era of immunotherapy has opened new avenues for the treatment of cancer patients; although, the benefits for CRC patients are still limited, which might—among other reasons—result also from a lack of appropriate predictive markers. Changes in the composition of immune cells in the tumor lesion may also affect the release of CTC into the blood. Microsatellite instability in CRC is a marker of immunogenicity and is associated with an increased abundance of tumor-infiltrating lymphocytes. Recently, Toh et al. found that microsatellite instability was associated with an increase in CTC numbers intra-operatively and post-operatively when combining data for all stage I–III CRC patients [60].

Functional CTC analysis using cell lines and xenograft models may also help to find appropriate targets or pathways for therapeutic intervention. Recent study results by Smit et al. showed that the PI3K/AKT/mTOR signaling pathway plays a key role in the proliferation of metastatic CRC [61]. They investigated a functional role of this pathway in a metastatic CRC cell line called CTC-MCC-41 and suggested that therapies targeting AKT and mTOR could be beneficial for targeting CTCs in CRC and possibly other tumor types [61]. Functional CRC models also provide a unique opportunity to study the biology of CTCs. In CRC, hierarchical organization is maintained during disease progression, and functional cancer stem cells are marked by Lgr5 expression. Fumagalli et al. aimed to investigate the cell of origin of metastases in CRC by using a mouse model of CRC and human tumor xenografts. Given that most disseminating cells were Lgr5− and could initiate metastatic growth, this leads to the assumption that the majority of metastases are seeded by Lgr5− cancer cells. Furthermore, the appearance of Lgr5+ CSCs is indispensable for the outgrowth of metastases founded by Lgr5− cancer cells. Their data indicate that besides targeting CSC and the CSC inducing niche, there is also a need to co-target endogenous cellular plasticity to inactivate any potential seeds of metastasis [62].

## 4. Clinical Applications of Circulating Cell-Free DNA

The quantity of ctDNA varies among individual patients and depends on the type and location of the primary or metastatic tumor lesion and the stage of the disease. The implementation of ctDNA in clinical practice holds great potential for early detection and personalized medicine in CRC [33,59]. The following chapter illustrates the latest clinical developments of using ctDNA as a biomarker in patients with CRC.

### 4.1. Early Detection of Cancer

ctDNA measurements hold promise for early detection in CRC and offer the possibility to address the heterogeneity of the tumor (Figure 1).

To encompass tumor heterogeneity, a complex blood test based on the detection of more than 1000 mutations in 16 cancer genes was combined with the measurement of eight tumor-associated blood plasma proteins. The so-called CancerSEEK-Test can detect CRC through assessment of the levels of circulating proteins and mutations in cell-free DNA and reached a sensitivity of more than 60% for CRC detection. The advantages of this test are the non-invasive screening by blood sampling (versus colonoscopy) and the low cost compared to the approved tests [63].

There is also a potential use of ctDNA methylation markers for early diagnosis of CRC. Luo et al. determine that a single ctDNA methylation marker, cg10673833, could yield high sensitivity (89.7%) and specificity (86.8%) for the detection of CRC and precancerous lesions in a high-risk population of 1493 participants in a prospective cohort study, which underlines the value of ctDNA methylation markers in the diagnosis, surveillance, and prognosis of CRC [31].

Future large-scale studies have to demonstrate that the ctDNA blood tests will add important information or easier acceptance by the individuals at risk than the established CRC screening tests including improved stool tests for occult blood and colonoscopy.

### 4.2. Assessment of Tumor Evolution towards Resistance to Therapy

The development of individualized treatment strategies might also profit from ctDNA analyses, in particular with regard to a better molecular understanding of resistance to therapy through ctDNA monitoring [64]. Despite a high degree of concordance between the mutational status of KRAS in tumor tissue and ctDNA [65,66], ctDNA can sometimes harbor KRAS mutations that are not detected in the primary lesion [25]. Sequential ctDNA analysis during EGFR inhibition has shown that KRAS and NRAS mutations can emerge rapidly due to the selective pressure exerted by targeted therapy [67]. Interestingly, the emerging population of KRAS-mutated subclones was able to decline after discontinuation of anti-EGFR therapy [67], indicating the potential to guide “cyclic therapy” characterized by sequential discontinuation and reintroduction of EGFR inhibitors based on ctDNA analyses. Patient-specific ctDNA assays can be developed through mutational analyses of primary tumors [68]. In addition, ctDNA analyses also helped to distinguish recurrent CRC from a second primary tumor [68].

ctDNA blood analysis can be complemented by tissue DNA analysis in case of a LB-negative result, which saves LB-positive patients from the unnecessary side effects of needle biopsies, and this strategy also appears to be a cost saving, in particular in the context of monitoring resistance to anti-EGFR-targeted therapies [69]. ctDNA genotyping has the potential to accelerate innovation in precision medicine and its delivery to individual patients. By evaluating the utility of ctDNA genotyping, Nakamura et al. enrolled 1687 patients with advanced gastrointestinal cancer and showed a significant shorter screening duration for patients undergoing ctDNA genotyping, which had a positive effect on trial enrollment without negative effects on treatment efficacy compared to tissue genotyping. Moreover, new candidates for potential clinical development were discovered through in-depth analysis of the ctDNA profiles [70].

### 4.3. Early Detection of Molecular Relapse by ctDNA Surveillance

Surveillance of ctDNA concentrations by sequential blood testing following the initial treatment (e.g., surgery, radiation, or (neo)adjuvant therapy) is another important clinical application of LB [71]. LB in early-stage, non-metastatic CRC must be sensitive enough to detect extremely low ctDNA levels. This challenge has been met by combining next-generation sequencing (NGS) and digital PCR (dPCR) to detect ctDNAs in non-metastatic CRC patients (*n* = 39); the NGS/dPCR test reached a sensitivity of 63.6% when combined with circulating carcinoembryonic antigen protein measurements [72]. ctDNA responders could be identified by monitoring ctDNA levels before and during chemotherapy including 1046 plasma samples from 230 patients with stage II colon cancer [73]. ctDNA analyzed by NGS was revealed post-surgery in 14 (7.9%) of 178 patients who did not receive adjuvant chemotherapy. Twenty-seven months later, ctDNA-positive patients had higher recurrence rates than ctDNA-negative patients, and a similar prognostic value was observed after completion of adjuvant chemotherapy [73].

Furthermore, ctDNA also identified patients at risk of developing metastases during neoadjuvant therapy and post-surgery. In the study of Khakoo et al., ctDNA detection rates were 74% (*n* = 35/47) before treatment, 21% (*n* = 10/47) at mid chemoradiotherapy (CRT), 21% (*n* = 10/47) after completing CRT, and 13% (*n* = 3/23) after surgery. Following 26.4 months of observation, ctDNA-positive patients had an unfavorable metastases-free survival [HR 7.1; 95% confidence interval (CI), 2.4–21.5; *p* < 0.001], as compared to ctDNA-negative patients [74]. In addition, a prospective multicenter trial that recruited 106 patients with locally advanced rectal cancer for treatment with nCRT followed by surgery ctDNA suggested that the median variant allele frequency of baseline ctDNA and ctDNA positivity at all four time points (baseline, during neoadjuvant CRT, pre-surgery, and post-surgery) is also a strong independent predictor of metastasis-free survival (*p* < 0.05) [75].

Taken together, these findings lead to the conclusion that ctDNA monitoring identified patients at risk of developing metastases during the neoadjuvant period and after surgery in CRC patients.

## 5. Conclusions and Perspectives

This review illustrates the latest developments in clinical applications of CTC and ctDNA as LB markers in CRC. LB enables the development of new methods for the early detection of primary cancer or minimal residual disease (MRD), monitoring the efficacy of cancer therapies, and determining therapeutic targets and resistance mechanisms to tailor therapy to the specific needs of an individual patient. Significant progress has been made in developing technologies to detect blood-based tumor-specific biomarkers, such as CTCs and ctDNA, and in developing downstream analyses of CTCs and ctDNA to provide new information about natural or therapy-induced tumor evolution in cancer patients. In addition, new members of the LB marker family include extracellular vesicles (EVs) [76], microRNAs [77], and tumor-derived platelets [78]. The newest findings have shown that miRNAs play an important role in many signal pathways. Dysregulated expression of several miRNA expressions is associated with a higher malignant potential and poor clinical response to therapy, and analysis of specific miRNA expression patterns can be used to predict chemotherapy efficacy [79,80,81]. miRNAs can also be detected in CTCs and contribute to a better understanding of the biology and clinical value of these cells [82]. Besides miRNA, increasing evidence has confirmed that EVs play a significant role in intercellular communication in CRC. EVs enable tumor communication and manipulation between tumor cells and the host immune system or the tumor microenvironment and can be induced by various cell signals such as hypoxia [83,84]. Both EVs and circulating miRNAs have great potential as biomarkers in cancer patients including CRC [77,78]. Besides tumor-derived cells and products, the peripheral blood is also a pool of host-derived cells (e.g., circulating immune cells, endothelial cells or fibroblasts) and cellular products (e.g., EVs from immune cells that may affect the immune response) [83]. Future studies on the interaction between CTCs and host cells might provide further insights into tumor biology with potential implications for the discovery of new prognostic and predictive biomarkers.

Immune checkpoint inhibition therapy has opened a new therapeutic avenue in oncology. However, only a fraction of patients will benefit so far from harnessing the immune response through the application of antibodies to inhibition checkpoint such as PDL1 or PD1, but the discovery of new checkpoints such as TGIT will offer new opportunities [85]. The utility of liquid biomarkers such as CTCs and ctDNA as prognostic and in particular predictive markers in the context of immunotherapies in solid tumors including gastrointestinal cancers have been recently reviewed in detail [86]. While ctDNA offers the possibility to determine the tumor mutational burden as potential (but still debated) predictive factor, CTC analysis can enlarge the spectrum by the detection of proteins relevant for the immune response such as MHC antigens or PDL1 on tumor cells responsible for the recognition or activation of T cells [87]. Interestingly, the expression of carcinoembryonic antigen and telomerase reverse transcriptase in CTCs predicted an unfavorable response to nivolumab, a PD1 inhibiting antibody [87].

To implement LB into clinical practice, harmonized protocols need to be developed. In this context, the EU-based CANCER-ID consortium has recently validated pre-analytical and analytical conditions of LB assays for CTCs [88], ctDNA [82], and microRNAs [83]. The activities of CANCER-ID are sustained by the new consortium designated European Society for LB (ELBS, www.elbs.eu, accessed on 4 September 2021), which is part of the International Alliance for LB Standardization [84].

Most importantly, the clinical utility of standardized LB assays needs to be proven in future interventional clinical trials. Previous studies have shown that CTC and ctDNA detection at the time of CRC diagnosis defines a subgroup of stage II patients at higher risk to develop relapse; however, it remains to be seen if these patients will benefit from more aggressive therapy. As another example, postoperative LB surveillance has been shown to be able to detect early molecular relapse many months before radiological imaging, but the key question is whether an earlier intervention based on the LB result leads to a survival benefit for CRC patients. Clinical trials addressing these (and other) relevant questions will open new avenues for introducing LB into future guidelines for the personalized treatment of CRC patients.

## Figures and Tables

**Table 1:** Overview of relevant studies for the detection of CTC in CRC using different isolation methods and their outcome. OS, overall survival. PFS, progression-free survival. HR, Hazard Ratio.CI, Confidence Interval.

Author Country (Year) [Reference]	Tumor Stage (UICC)	Number of Patients and Detection Rate n, (Percentage%)	Method	Sampling Time	Clinical Outcome
Abdalla et al. Germany 2021 [26]	I–IV data	68, 31 (45%) preoperatively	Cellsearch^®^data	pre- and postoperative	Multivariate analyses showed that only preoperative detection of ≥1 CTCs/7.5 mL is an independent prognostic indicator for OS (HR 3.14; CI 1.18–8.32; *p* = 0.021).
Silva et al. Brazil 2021 [36]	IV	75, 45 (60%)	ISET^®^	pretherapeutic	In multivariate analysis, presence of ≥1.5 CTCs/mL was associated with worse OS (HR 2.34, CI 1.11–4.9, *p* = 0.025).
Kust et al. Croatia 2016 [37]	I–III	82, 69 (72.6%) preoperatively 74 (77.9%) postoperatively	RT-PCR	preoperative and postoperative	PFS was significantly shorter in patients with CK20-positive CTCs postoperatively in comparison to patients negative for CK20 postoperatively (*p* = 0.01, log-rank test). CTC detection was not significant in multivariate analysis outcome.
Sotelo et al. Spain 2015 [38]	I–III	519, 166 (35%)	Cellsearch^®^	postoperative and pretherapeutic	≥1 CTCs/7.5 mL was not associated with worse PFS (HR 0.97, CI 0.68–1.38, *p* = 0.85) or OS (HR 1.03, CI 0.66–1.59, *p* = 0.89).
Bork et al. Germany 2015 [34]	I–IV	287, 30 (10.5%)	Cellsearch^®^	pre- and postoperative	Multivariate analysis showed that preoperative detection ≥ 1 CTCs/7.5 mL was associated with worse OS (HR 5.5; CI 2.3–13.6; *p* = 0.001) and PFS (HR 12.7; CI 5.2–31.1; *p* = 0.001) in stage I–III CRC as well as worse OS (HR 5.6; CI I2.6– 12.0; *p* = 0.001) and PFS (HR 7.8; CI 3.9–15.5; *p* = 0.001) in stages I–IV.
Seeberg et al. Norway 2015 [39]	IV	194, 37 (19.6%)	Cellsearch^®^	preoperative	In multivariate analysis, the presence of ≥2 CTCs/7.5 mL at baseline was associated with worse PFS (HR 2.32, CI 1.26–4.27, *p* = 0.007) and OS (HR 2.48, CI 1.40–4.38, *p* = 0.002)
Yokobori et al. Japan 2013 [40]	I–IV	711, 179 (33.6%)	RT-PCR	preoperative	Multivariate analysis showed that PLS3-positive CTCs are associated with poor OS (HR 2.17; CI 1.38–3.40) and PFS (HR 2.32; CI 1.42–3.74).
Cohen et al. US, Netherlands, and UK 2008 [41]	IV	430, ≥1 CTCs,198 (48%). ≥3 CTCs, 108 (26%)	Cellsearch^®^	Pre- and post-therapeutic	In multivariate analyses, patients with ≥3 CTCs/7.5 mL at baseline had shorter PFS (HR 1.74, CI 133–2.26, *p* ≤ 0.001), and OS (HR 2.45, CI 1.77–3.39, *p* ≤ 0.001).

## Abbreviations

cfDNA	circulating free DNA
CI	confidence interval
ctDNA	circulating tumor-derived DNA
CTC	circulating tumor cell
CRC	colorectal cancer
CRT	chemoradiotherapy
DEP	dielectrophoresis
DFS	disease-free survival
EGFR	epithelial growth factor receptor
EMT	epithelial-mesenchymal transition
EpCAM	epithelial cell adhesion molecule
EPISPOT	epithelial immune SPOT
EV	extracellular vesicle
FOLFOX	folinic acid, fluorouracil and oxaliplatin
FOLFOXIRI	fluorouracil, folinate, oxaliplatin, and irinotecan
HR	hazard ratio
HER2	human epidermal growth factor receptor 2
LB	liquid biopsy
MRD	minimal residual disease
mRNA	messenger RNA mRNA
NGS	next generation sequencing NSCLC, non-small cell lung carcinoma
OS	overall survival
PFS	progression-free survival
rtPCR	real-time polymerase chain reaction
tDNA	tumor DNAs
WGA	whole genome amplification
